# Liver and Skeletal Muscle Metabolome Characterization in Peripartal Dairy Cows Fed Rumen-Protected Methionine or Rumen-Protected Choline

**DOI:** 10.3390/ani16050705

**Published:** 2026-02-24

**Authors:** Valentino Palombo, Zheng Zhou, Lam Phuoc Thanh, Mariasilvia D’Andrea, Daniel N. Luchini, Juan J. Loor

**Affiliations:** 1 Dipartimento di Agricoltura, Ambiente e Alimenti, Università degli Studi del Molise, 86100 Campobasso, Italy; valentino.palombo@unimol.it (V.P.);; 2 Department of Animal Sciences, Michigan State University, East Lansing, MI 48824, USA; 3 Faculty of Animal Sciences, College of Agriculture, Can Tho University, Can Tho 900000, Vietnam; 4 Adisseo, Alpharetta, GA 30022, USA; 5 Department of Animal Sciences, Division of Nutritional Sciences, University of Illinois, Urbana, IL 61801, USA

**Keywords:** periparturient dairy cows, rumen-protected methionine, rumen-protected choline, metabolomics, transition cow

## Abstract

The transition period in dairy cows involves profound metabolic adaptations that challenge energy balance and liver function. This study evaluated the effects of rumen-protected methionine (RPM) and choline (RPC) on hepatic and skeletal muscle metabolism. Twenty-one multiparous Holstein cows from a 2 × 2 factorial design (CON, RPM, RPC) underwent liver and semitendinosus biopsies at −10, +7, and +20 d relative to parturition. Untargeted LC-MS metabolomics detected 2288 and 1454 molecular features in liver and muscle. Data were analyzed using mixed-model ANOVA (FDR ≤ 0.05), complemented by multivariate approaches including sparse PLS-DA and PERMANOVA to assess global metabolic variation. Metabolite annotation was performed using HMDB (±0.005 Da). Dietary supplementation significantly affected 105 hepatic metabolites, whereas time influenced 552 metabolites, generally reflecting increases or decreases in concentration from the prepartum to early postpartum periods. Network analysis identified nine hepatic co-expression modules associated with RPM and RPC. Hub metabolites included glucose-6-phosphate, mannose-6-phosphate, and sphingomyelins, indicating modulation of carbohydrate and lipid metabolism. In muscle, treatment effects were modest, with PERMANOVA and PLS-DA confirming limited discrimination among groups and a predominant temporal effect. Overall, RPM and, to a lesser extent, RPC modulated key hepatic metabolic pathways, supporting energy and redox homeostasis during early lactation. These findings highlight the potential of methyl-donor supplementation to enhance metabolic resilience at the tissue level in transition cows.

## Simple Summary

This study used untargeted metabolomics of liver and muscle to explore the effects of rumen-protected methionine and choline during the periparturient period in dairy cows. During the physiological challenges of early lactation, these methyl donors were associated with changes in metabolites involved in the glucose, lipid, and redox-related pathways, particularly in the liver. Network-based analyses identified groups of correlated metabolites potentially linked to dietary treatments, providing insights into tissue-specific metabolic responses. Overall, these findings contribute to a better understanding of how targeted nutritional strategies may influence metabolic adaptation in transition cows, with possible implications for animal health and production efficiency.

## 1. Introduction

The periparturient period in dairy cows, spanning three weeks before to three weeks after parturition, is characterized by profound metabolic and physiological adaptations. These changes compromise immune function, increasing susceptibility to diseases such as mastitis, metritis, and ketosis [1,2]. The heightened nutrient demands for fetal growth and lactation, particularly for energy (glucose, fatty acids) and protein (amino acids), coupled with a decline in voluntary feed intake before calving, induce substantial metabolic stress. Consequently, cows experience a negative energy and protein balance postpartum, which contributes to an increased incidence of disease and reduced production efficiency [3,4]. Feeding rumen-protected methionine (RPM) and choline (RPC) can help alleviate nutrient insufficiency and metabolic stress during this critical period [5,6]. These nutrients play pivotal roles in metabolism, with methionine serving as a precursor for other sulfur-containing amino acids and essential methylation reactions. As a limiting amino acid, methionine significantly influences milk protein synthesis and overall lactation performance, with studies demonstrating its positive impact on milk production [5,7]. Mechanistically, methionine donates methyl groups via S-adenosylmethionine (SAM), supporting transmethylation reactions critical for protein and DNA methylation as well as the synthesis of antioxidants such as glutathione [8]. Similarly, choline contributes to phosphatidylcholine biosynthesis, essential for hepatic very-low-density lipoprotein (VLDL) formation and lipid export [9], while also supporting DNA methylation [10] and cell membrane integrity [11]. RPC supplementation during the transition period has been associated with improved dry matter intake, postpartal milk yield, and fat-corrected milk production [12,13]. Beyond their metabolic roles in peripartal dairy cows, RPM and RPC have garnered attention for their potential to modulate immune responses, reduce oxidative stress, and mitigate inflammatory processes [14,15,16,17]. Thus, RPM and RPC offer beneficial nutritional solutions for the management of transition dairy cows.

Given the interconnected roles of methionine and choline in multiple metabolic pathways, a systems-level approach is required to comprehensively characterize their biological effects during the transition period. In this context, metabolomics has emerged as a powerful tool for capturing global metabolic responses to dietary interventions [18]. A recent review also emphasized the value of metabolomics for unraveling the etiopathology of transition disorders such as ketosis, highlighting its potential to identify novel diagnostic biomarkers and improve disease prevention strategies [19].

Among the key metabolic tissues, the liver and skeletal muscle are directly influenced by nutrient supply, impacting energy metabolism and overall health [20,21] and were selected because they provide complementary insights into systemic metabolic adaptations during the peripartal period. The liver acts as a central hub for nutrient metabolism, detoxification, and lipid export, whereas skeletal muscle represents a major site for amino acid utilization and energy storage. Therefore, simultaneous profiling of these tissues may provide an integrated view of systemic metabolic adaptations to methyl donor supplementation, potentially capturing both hepatic adjustments and peripheral nutrient redistribution. Despite growing interest in metabolomics for understanding metabolic adaptations in dairy cows, no studies have yet characterized the longitudinal metabolome in the liver and skeletal muscle in response to feeding RPM or RPC. Previous studies using similar experimental cohorts [6,7,22,23,24] have focused on production performance, immune or transcriptional responses, a limited number of metabolic markers, or single tissues, leaving a gap in understanding tissue-specific and systemic metabolic adjustments across the peripartal period.

In this study, we used untargeted metabolomic profiling to explore the effects of RPM and RPC on liver and skeletal muscle collected throughout the periparturient period [24]. We hypothesize that supplementation with RPM and RPC could modulate hepatic and muscle metabolic profiles, enhancing pathways related to energy metabolism, lipid export, and antioxidant capacity, and that these effects will vary over the periparturient period.

## 2. Materials and Methods

### 2.1. Animals and Experimental Design

All procedures for this study were conducted in accordance with a protocol (no. 13023) approved by the Institutional Animal Care and Use Committee of the University of Illinois at Urbana–Champaign (UIUC). The experiment was conducted at the UIUC Dairy Research Farm, and the cows used were a subset from a previous experiment [24]. In the main experiment, a total of 81 cows were used in a randomized, complete, unbalanced block design with a 2 × 2 factorial arrangement of methionine (RPM) and choline (RPC) level (with or without). Cows were blocked by expected calving date, and within each block, they were balanced for parity, previous lactation milk yield, and body condition score. Minor deviations in enrollment and health-related removals resulted in a slightly unbalanced design. Treatments included: control (CON, n = 20) with no supplementation; RPM (Smartamine—M, Adisseo NA, Alpharetta, GA, USA; n = 21) at 0.08% of dietary dry matter; RPC (ReaShure—Balchem Inc., New Hampton, NY, USA; n = 20) at 60 g/d; and a combination of RPM + RPC (MIX, n = 20). Both RPM and RPC were top-dressed once daily from −21 ± 2 days relative to expected calving through 30 days in milk. For the metabolomics analysis presented in this study, a subset of 21 cows was selected (7 cows per treatment; CON, RPM, and RPC). The MIX group was not included in the metabolomics subset. This decision was made to specifically disentangle the individual metabolic effects of methionine and choline supplementation during the peripartal period. Including the combined treatment would have increased the complexity of metabolite interpretation due to potential additive or interactive effects between the two methyl donors.

### 2.2. Liver and Skeletal Muscle Biopsies

From the larger experimental cohort, seven cows per treatment group with a complete set of biopsies were randomly selected for metabolomic analysis, resulting in a total of 63 samples (7 cows × 3 treatments × 3 time points). Liver tissue was collected at −10, +7, and +20 d relative to parturition via puncture biopsy, as described by [6]. Skeletal muscle biopsies were taken from the semitendinosus muscle at −10, +7, and +21 d relative to parturition using a needle biopsy instrument, as described by Thanh et al. [25]. On average, prepartum samples were harvested at −10.9 ± 1.3 days relative to actual calving date. Liver and muscle tissue were snap-frozen in liquid nitrogen and then stored at −80 °C. Approximately 100 mg of liver and muscle tissue was extracted 1 mL 70% methanol using a Polytron homogenizer (Kinematica Inc., Bohemia, NY, USA) prior to delivery to the UIUC Carver Metabolomics Core, Roy J. Carver Biotechnology Center, Urbana.

### 2.3. Untargeted Metabolite Fingerprinting and Data Processing

Samples were initially spiked with a mixture of deuterium-labeled, stable isotope-labeled, and non-labeled surrogate internal standards. One µL of each processed sample was injected into the instrument. Samples were analyzed using a Dionex Ultimate 3000 series UHPLC system (Thermo Fisher Scientific, Waltham, MA, USA) coupled with a Q-Exactive mass spectrometer (Thermo Fisher Scientific, Waltham, MA, USA), as previously described [26]. Reversed-phase liquid chromatography (RPLC) was performed using a Waters Acquity ethylene-bridged hybrid C18 column (100 mm × 2.1 mm; 1.7 μm Waters Corp, Milford, MA, USA); the column was maintained at 30 °C with a flow rate of 0.3 mL/min. The mobile phases consisted of water with 0.1% formic acid and acetonitrile with 0.1% formic acid with gradient. Data were acquired in both positive and negative ionization modes. All LC-MS raw data files were processed using MS-DIAL version 4.90 for peak detection, alignment, adduct annotation, and compound identification [27]. Key parameters included: MS1 tolerance of 0.005 Da, MS2 tolerance of 0.01 Da, minimum peak height of 10,000 (negative mode) and 100,000 (positive mode), mass slice width of 0.05 Da, linear weighted moving average smoothing with a smoothing level of 3 scans, and a minimum peak width of 5 scans. Adduct settings included [M−H]^−^ and [2M−H]^−^ for negative mode, and [M + H]^+^, [2M + H]^+^, [M + Na]^+^ for positive mode. Peak alignment was performed using the Retention Time Tolerance algorithm in MS-DIAL with automatic retention time correction across all samples. All peak intensities were normalized to the average peak height of the corresponding internal standards for each analysis (RPLC-Positive and RPLC-Negative). No additional batch correction was applied, as samples were processed in a single analytical run under consistent instrument conditions. A total of 4218 molecular features were initially detected in liver samples and 1960 in muscle. After data preprocessing, which included the removal of artifacts, rare features, and features with excessive missing values (>50%), the final datasets consisted of 2288 liver and 1454 muscle molecular features for downstream analyses. Missing values were imputed by replacing them with 1/5 of the minimum positive value of their corresponding variables, and log_10_ transformation and autoscaling were applied using MetaboAnalyst v6.0 software [28].

### 2.4. Univariate Analysis and Metabolite Annotation

Statistical differences between the experimental groups were examined using mixed ANOVA, also known as split-plot ANOVA, in the R environment [29], including treatment, time, and treatment × time as fixed effects, whereas cow was the random effect. Metabolites were deemed significant at an FDR ≤ 0.05 threshold. For metabolite identification, significant metabolites were first manually annotated by matching their average mass weight values against the HMDB database within a tolerance range of ±0.005 Da. A broader threshold of ±0.05 was applied in cases where no matches were obtained with the preferential and most stringent criterion. To increase robustness, a secondary verification was performed using the R package *MetaboAnnotation* (v1.4.3) [30], specifically employing the function *Mass2zParam* and *matchValues*, again with HMDB as the reference database. Only metabolites consistently annotated by both approaches were retained for downstream analyses. Metabolite set enrichment analysis was performed using MBRole (v3.0) software [31] to highlight key pathways and biological patterns. For each tissue, analyses were performed separately on treatment- and time-associated metabolites.

### 2.5. Network Analysis of Metabolite Co-Expression

To better understand the metabolite network organization, a weighted correlation network analysis (WGCNA) was performed. This method is a network inference algorithm derived from a biological profile and is widely applied for studying biological networks [32,33]. This algorithm relies on the pairwise correlation between metabolites and provides information such as network module (a subset of metabolites that highly correlate each other) and eigen-metabolite (an imaginary metabolite that represents a module). The analysis was performed with the *WGCNA* (v1.72-5) package in R [34] considering log_10_ transformations and auto scaling data, and corrected for the time effect with the *limma* (v3.56.2) R package [35]. A similarity matrix was constructed to calculate the Pearson correlation coefficients between metabolite pairs. This matrix was then converted into an adjacency matrix, applying a soft threshold power (β) to enhance strong correlations while minimizing weak ones. The optimal β value was determined by evaluating values ranging from 1 to 50, selecting the smallest β at which the correlation coefficient reached ≥ 0.90, while balancing the mean connectivity drop, ensuring scale-free network properties. Next, the adjacency matrix was transformed into a topological overlap matrix, representing the strength of metabolite associations. Clustering was performed using the *DynamicTreeCut* algorithm, allowing for the identification of modules (clusters of co-expressed metabolites). Modules were merged based on eigengene similarity (threshold > 0.25), with a minimum module size of 30 metabolites. The *flashClust* function was used to assess co-expression similarity among modules. The module eigengene, representing the first principal component of each module, was calculated as the weighted average expression of all metabolites within the module.

### 2.6. Hub Metabolite Identification

To identify hub metabolites, Maximal Clique Centrality (MCC) scores were computed using the *cytoHubba* (v0.1) plugin [36] within Cytoscape (v3.9.1) [37]. Metabolites with the highest MCC scores were designated as hub genes, as they exhibited the strongest interconnections within their respective co-expression modules, suggesting key functions [38,39]. To further validate these findings, we evaluated the correlation between module eigengenes and experimental traits, defining hub metabolites as those with an absolute module membership (MM) greater than 0.6 and an absolute gene significance (GS) greater than 0.6 [40]. Although the term gene significance is traditionally used in WGCNA frameworks developed for transcriptomic studies, in this metabolomics-based analysis, it refers to the strength of association between each metabolite and the trait of interest. Therefore, GS here represents metabolite-trait significance, avoiding any implication of gene-level regulation. Since module eigengenes represent the main expression pattern of their constituent metabolites, a high correlation between MM and GS supports a strong and reliable association between the identified modules and the biological traits under investigation [41,42].

### 2.7. Multivariate Analysis and Model Validation

To complement univariate and network-based analyses, Partial Least Squares Discriminant Analysis (PLS-DA) was performed to assess the global effect of treatment on metabolite profiles. Normalized metabolite concentrations were used as predictor variables, while treatment groups (CON, RPM, and RPC) were used as the response variable. PLS-DA was conducted using the *mixOmics* (v6.24.0) R package [43], and model tuning was performed with the sparse PLS-DA function considering 5 components, 5-fold cross-validation repeated 50 times, maximum distance as classification metric, and Balanced Error Rate (BER) to select the optimal number of variables. Model performance was assessed through cross-validated error rates, and variable importance in projection (VIP) scores were computed to identify metabolites contributing most to group discrimination. The top 10 metabolites ranked by VIP in the first component were extracted.

To statistically validate global multivariate effects, a Permutational Multivariate Analysis of Variance (PERMANOVA) was performed in the R environment using the *vegan* (v2.6-4) R package. Euclidean distances were calculated on log_10_-transformed and autoscaled metabolite data, and the model tested the effects of treatment, time, and their interaction using 999 permutations. PERMANOVA provided a complementary statistical assessment of whether the overall metabolite profiles significantly differed between groups, corroborating the separations observed in PLS-DA. This integrated multivariate framework allowed for visualization of group separation, the identification of discriminant metabolites, and confirmation of the overall treatment and time effects.

## 3. Results

Physiological responses at the liver tissue and plasma level due to feeding RPM or RPC have been published in a series of manuscripts and will not be recapitulated here. The reader is referred to Zhou et al. [6,22,23] and Vailati-Riboni et al. [7].

### 3.1. Metabolite Profiling and Differential Expression in Liver

The raw dataset consisted of 2288 molecular features across 63 samples (7 cows per treatment: CON, RPM, RPC; three time points: −10, +7, +20 days relative to parturition). Missing values accounted for 15.8% (26,061 values) and subsequently imputed. For the Treatment effect, 138 molecular features were significant (FDR ≤ 0.05; Table S1), whereas 1586 features varied significantly over time (FDR ≤ 0.05). Annotation using the HMDB library (±0.005 Da) and verification with the *MetaboAnnotation* R package resulted in 105 unique metabolites for treatment-associated features (corresponding to 20 of the 138 molecular features; Table S2) and 552 metabolites for time-associated features (representing 202 of the 1586 molecular features; Table S3). The remaining features did not yield HMDB annotations.

**Figure 1:**
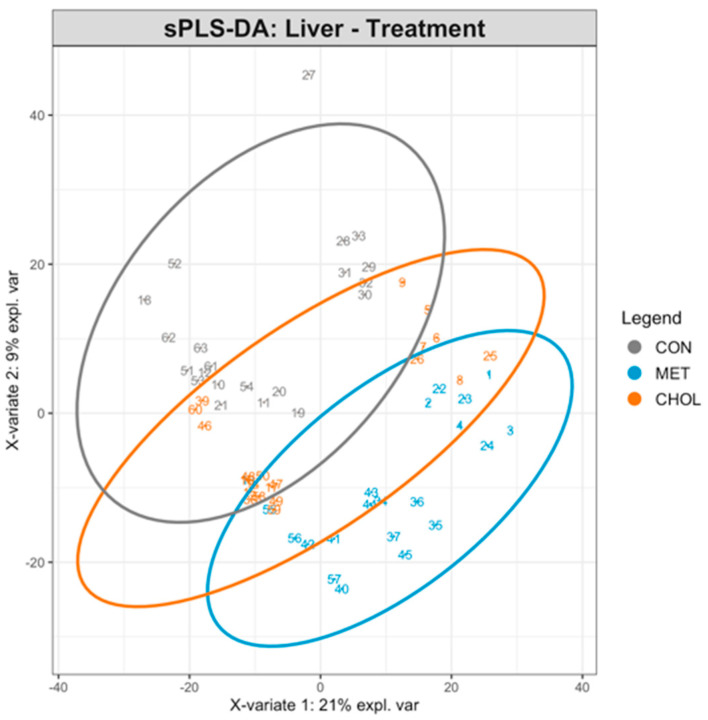
Sparse Partial Least Squares Discriminant Analysis (sPLS-DA) of liver metabolome in Holstein cows fed rumen-protected methionine (RPM) or choline (RPC) during the close-up (−10 days relative to parturition) and early lactation (+7 and +20 days in milk) periods. Each point represents an individual liver sample, with 7 cows per treatment at each time point (total 21 samples per treatment group), projected on the first two sPLS-DA components. Samples are colored according to treatment groups (CON = control, MET = RPM, CHOL = RPC). Ellipses represent 95% confidence intervals for each group. The plot highlights the separation of metabolite profiles between treatment groups, indicating treatment-specific effects on liver metabolism.

Complementary multivariate analysis using PLS-DA indicated that treatment groups were partially separable based on global liver metabolite profiles (Figure 1). Cross-validation indicated a decrease in classification error with increasing components, with the best model achieving an overall balanced error rate (BER) of 0.2987 on four components, suggesting moderate discrimination among the CON, RPM, and RPC groups. The top 10 VIP metabolites on the first component included six molecular features (i.e., M355, M740, M843, M1214, M1763, M161) also identified by ANOVA (Table S1), reinforcing their potential biological relevance.

The HMDB-identified metabolites for treatment (105 compounds) and time (552 compounds) effects were subjected to KEGG pathway enrichment analysis (Table 1 and Table 2) using MBRole3 with *Bos taurus* as the reference. For treatment, 21 pathways were significantly overrepresented (FDR ≤ 0.05; Table 1), including Fructose and mannose metabolism, Inositol phosphate metabolism, and Galactose metabolism, Amino sugar and nucleotide sugar metabolism, Glucagon signaling pathway, Phosphatidylinositol signaling system, Glycolysis/Gluconeogenesis, and Starch and sucrose metabolism. For the time effect, 14 pathways were enriched (FDR ≤ 0.05; Table 2), notably Amino sugar and nucleotide sugar metabolism, and Fructose and mannose metabolism.

### 3.2. Co-Expression Network Analysis and Hub Metabolite Identification in Liver

**Figure 2:**
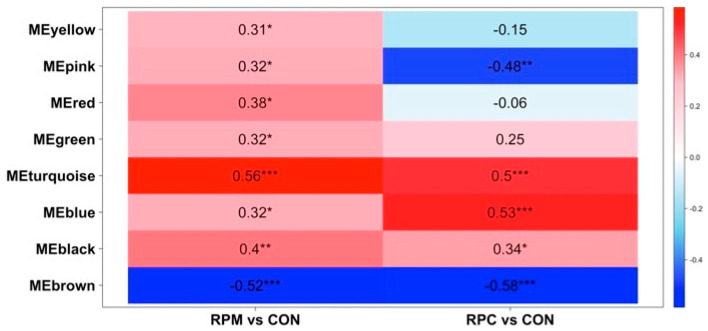
Heatmap of the correlation between module eigengenes (ME) and the treatment conditions in the liver dataset of Holstein cows fed rumen-protected methionine (RPM) or choline (RPC) during the close-up (from −10 d to parturition) and early lactation (from +7 to +20 days in milk) periods. Each row corresponds to a ME where the module name (e.g., MEyellow, MEpink) refers to the color-coded module identified by WGCNA and represents the first principal component summarizing the molecular features within that module. Each cell reports the correlation coefficient and the corresponding *p*-value. CON stands for control group. Levels of statistical significance are indicated as follows: *** *p* ≤ 0.001, ** *p* ≤ 0.01, * *p* ≤ 0.05.

A total of 2288 molecular features from the 63 liver samples, after missing value imputation, were retained for co-expression analysis. The adjacency matrix was constructed using a β of 3, selected based on the scale-free topology criterion (fit index = 0.9; Figure S1), confirming the appropriateness of this parameter. Modules were defined with a dissimilarity threshold (MEDissThres) of 0.25, resulting in nine co-expression modules (Figure S2). Metabolites not assigned to any module were grouped into a gray module, which was excluded from further analysis. The identified modules were largely independent, reinforcing the robustness of the clustering process. Correlations between module eigengenes and experimental contrasts varied across modules, with all liver-derived modules (Figure 2) having statistically significant associations (*p* ≤ 0.05). Turquoise, brown, and blue modules exhibited moderate-to-high correlations (|correlation coefficient| ≥ 0.5, *p* ≤ 0.001; Figure 2) and were selected for further analyses.

**Figure 3:**
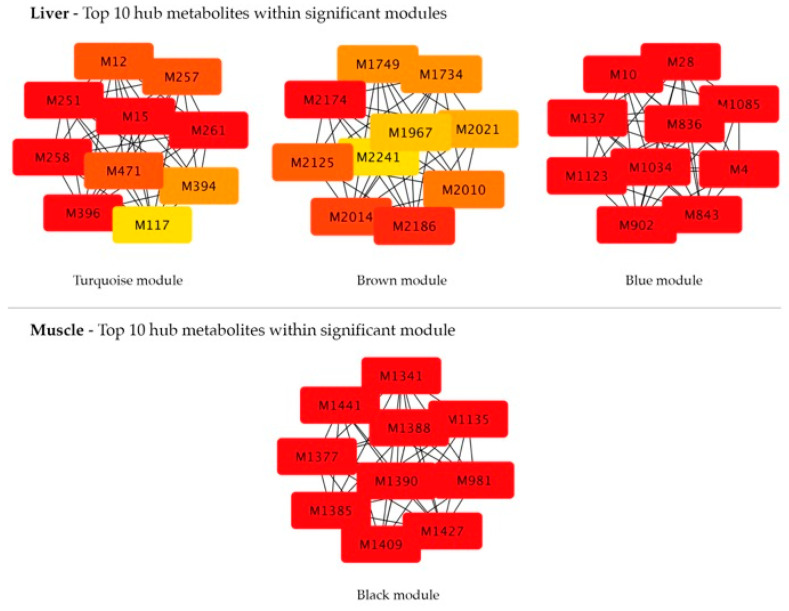
Network representation of the top 10 hub molecular features within significant modules identified in liver and muscle tissues using the Maximal Clique Centrality (MCC) approach in Cytoscape.

Metabolite co-expression networks were then constructed for each candidate module, where nodes represent metabolites and each edge represents pairwise correlations. Hub metabolites, defined as highly connected nodes, were identified using the MCC approach via the *CytoHubba* plugin in Cytoscape. The top ten hub metabolites per module are reported in Table 3 and Figure 3. Hub candidates were further validated using the MM–GS approach (|MM| > 0.6 and |GS| > 0.6), and those consistently identified by both methods were highlighted as most robust hub candidates (Table 3). Considering a total of 30 hub metabolites (i.e., top 10 for each module of interest), 19 were matched to HMDB entries. Overall, 167 unique compounds were retained, representing metabolites consistently identified by manual HMDB annotation and the *MetaboAnnotation* package using a ±0.05 Da mass tolerance (Table S4), since a more stringent ±0.005 Da tolerance yielded insufficient annotations.

### 3.3. Metabolite Profiling and Differential Expression in Muscle

In muscle, the raw dataset consisted of 1454 molecular features across 63 samples. A total of 13,706 missing values (15%) were identified and subsequently imputed. For the treatment effect, only one molecular feature was significant at FDR ≤ 0.05, while 31 metabolites reached significance at an uncorrected *p*-value ≤ 0.01 (Table 4) and were retained for further analysis. Based on their average molecular weights (tolerance of ±0.005 Da), 15 candidate compounds were identified in the HMDB library using both manual curation and the *MetaboAnnotation* package (Table 5), with five out of the initial 31 features matched to HMDB entries. For the time effect, 275 metabolites were significant at FDR ≤ 0.05 (Table S5), yielding 97 unique HMDB candidate compounds (Table S6). A total of 64 out of the initial 275 molecular features could be matched to HMDB entries. Pathway enrichment analysis did not yield any significant results.

**Figure 4:**
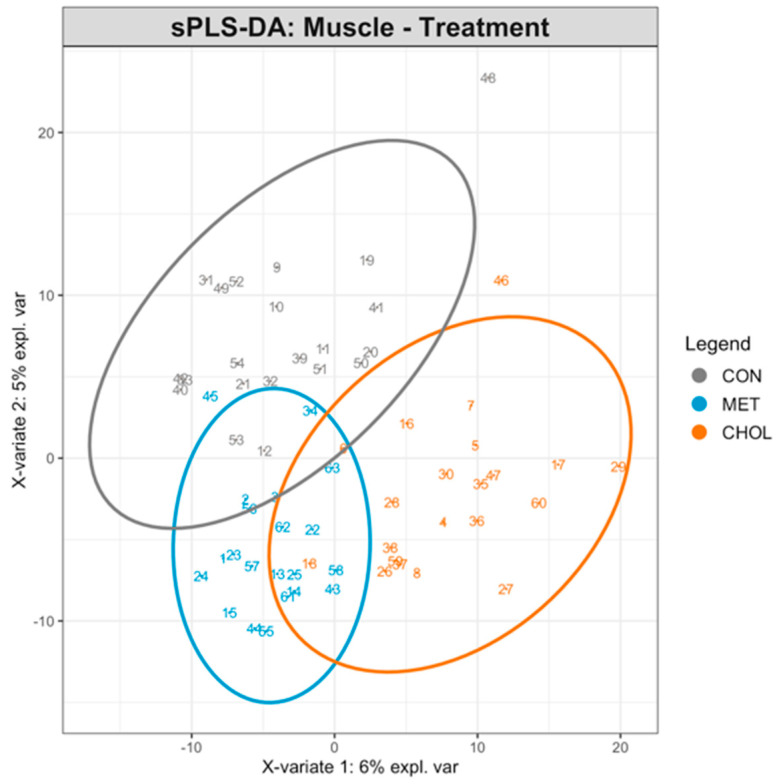
Sparse Partial Least Squares Discriminant Analysis (sPLS-DA) of liver metabolome in Holstein cows fed rumen-protected methionine (RPM) or choline (RPC) during the close-up (−10 days relative to parturition) and early lactation (+7 and +20 days in milk) periods. Each point represents an individual muscle sample, with 7 cows per treatment at each time point (total 21 samples per treatment group), projected on the first two sPLS-DA components. Samples are colored according to treatment groups (CON = control, MET = RPM, CHOL = RPC). Ellipses represent 95% confidence intervals for each group. The plot highlights the separation of metabolite profiles between treatment groups, indicating treatment-specific effects on muscle metabolism.

To further explore global metabolic patterns associated with treatment, sPLS-DA was performed, but the model showed limited discriminative ability between groups (BER = 0.61 for the first component, 0.47 for the second component; Figure 4), consistent with the univariate analysis and indicating a weak treatment-driven separation relative to the strong temporal effect. PERMANOVA confirmed significant effects of treatment (R^2^ = 0.044, *p* = 0.001), time (R^2^ = 0.078, *p* = 0.001), and their interaction (R^2^ = 0.078, *p* = 0.002) on the global muscle metabolome, although the proportion of explained variance was low, with time accounting for the largest share of variability, confirming its predominant role. The majority of the variance remained unexplained (residual R^2^ = 0.80), indicating substantial inter-individual variability.

Notably, six out of the top 10 VIP molecular features (i.e., M137, M339, M395, M438, M1044, and M1303) overlapped with those identified by univariate ANOVA (uncorrected *p*-value ≤ 0.01; Table 4). This concordance between multivariate and univariate approaches strengthens the evidence that these metabolites represent robust treatment-associated molecular features, highlighting their potential biological relevance despite the overall modest treatment effect.

### 3.4. Co-Expression Network Analysis and Hub Metabolite Identification in Muscle

**Figure 5:**
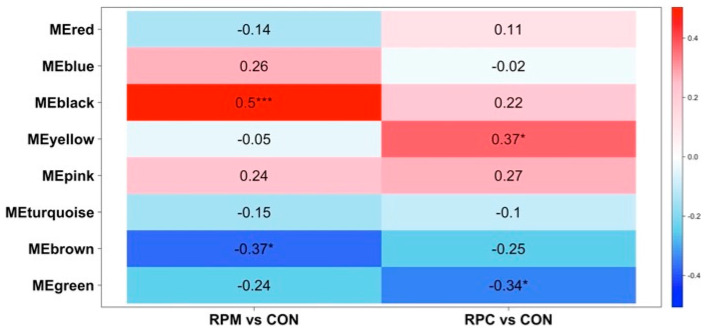
Heatmap of the correlation between module eigengenes and the treatment conditions in the muscle dataset of Holstein cows fed rumen-protected methionine (RPM) and choline (RPC) during the close-up (from −10 d to parturition) and early lactation (from +7 to +20 days in milk) periods. Each row corresponds to a ME where the module name (e.g., MEyellow, MEpink) refers to the color-coded module identified by WGCNA and represents the first principal component summarizing the molecular features within that module. Each cell reports the correlation coefficient and the corresponding *p*-value. CON stands for control group. Levels of statistical significance are indicated as follows: *** *p* ≤ 0.001, * *p* ≤ 0.05.

A total of 1454 molecular features, obtained after missing value imputation, were retained for co-expression analysis. The adjacency matrix was constructed using a β of 3, selected according to the scale-free topology criterion (fit index = 0.9; Figure S3). Module detection using a MEDissThres of 0.25 identified nine co-expression modules (Figure S4). Metabolites not assigned to any module were grouped in the gray module and excluded from downstream analysis. Module-trait correlation analysis revealed four modules significantly associated with experiment contrasts (*p* ≤ 0.05; Figure 5) among which the black module showed a moderate correlation (|correlation coefficient| = 0.5, *p* ≤ 0.001; Figure 5). Subsequent analyses therefore focused only on this module. Hub metabolites were identified using the MCC approach (Table 6 and Figure 3); however, none of the top-ranked features met the MM–GS significance criteria (|MM| > 0.6 and |GS| > 0.6). Annotation of the top ten MCC-ranked molecular feature yielded 28 unique HMDB compounds based on average molecular weights matching with a tolerance of ±0.05 Da (Table S7).

## 4. Discussion

### 4.1. Metabolic Adaptations in Liver Due to RPM and RPC

**Figure 6:**
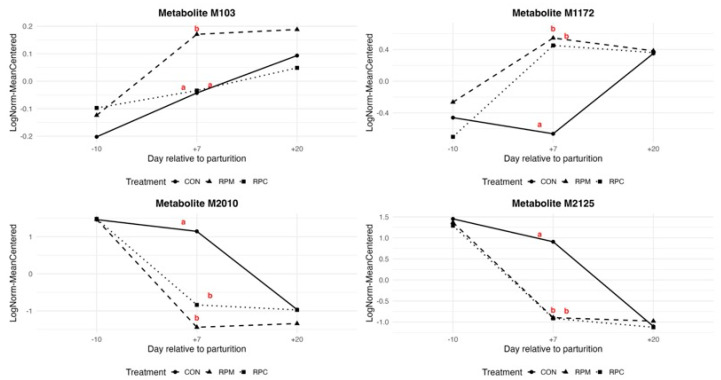
Levels of four selected molecular features in the liver tissue of Holstein cows fed rumen-protected methionine (RPM) or choline (RPC) during the close-up (from −10 d to parturition) and early lactation (from +7 to +20 days in milk) periods. CON stands for control group. Different letters indicate significant differences among treatments (adjusted *p*-value ≤ 0.05). The selected molecular features correspond to: M103 (putatively annotated as glucose 6-phosphate, glucose 1-phosphate, fructose 6-phosphate, or β-D-fructose 6-phosphate), M1172 (6-formylpterin), M2010 (phosphatidylethanolamine derivatives PE(18:2(9Z,12Z)/LTE4) and PE(LTE4/18:2(9Z,12Z))), and M2125 (sphingomyelins SM(d18:1/20:1(11Z)) and SM(d18:2(4E,14Z)/20:0)). Tentative metabolite annotations are reported in Table S2.

Our metabolomics results suggest a potential influence of RPM and RPC in modulating key metabolic pathways such as glycolysis/gluconeogenesis, the pentose phosphate pathway, AMPK signaling, and inositol phosphate metabolism (Table 1). This conclusion is based on metabolites identified as treatment-associated either by univariate ANOVA (FDR ≤ 0.05) or by PLS-DA VIP scores, followed by annotation using HMDB. Collectively, these pathways are central to hepatic energy metabolism and redox balance during the peripartal period. Among the treatment-associated metabolites detected in liver, the molecular feature M103 emerged as particularly noteworthy. This metabolite displayed a relatively greater concentration at day +7 in the RPM group compared with other groups (Figure 6), representing the most evident treatment-specific response within the metabolomics dataset. Several key metabolites with molecular weights within a ±0.005 Da tolerance range were identified as putative matches for M103, including glucose 6-phosphate, glucose 1-phosphate, fructose 6-phosphate, and beta-D-fructose 6-phosphate (Tables S1 and S2). These metabolites are central to glycolysis and gluconeogenesis, processes essential for glucose homeostasis [44], including milk synthesis.

Consistent with this interpretation, previous studies have reported increased hepatic glucose release during the early postpartal period, coinciding with enhanced net amino acid uptake by the liver [6,45]. Given the metabolic shift necessary to maintain energy balance during early lactation, it is possible that RPM plays a role in supporting glucose availability, e.g., by optimizing DMI, as reported in the original manuscript from this study [22]. Furthermore, the presence of mannose 6-phosphate, a precursor of fructose 6-phosphate and an entry substrate for glycolysis [46], suggests a potential adaptation favoring continuous energy supply through glycolysis.

Beyond individual metabolites, the pathway enrichment analysis (Table 1) highlighted the involvement of insulin resistance and glucagon signaling, two key regulators of glucose and lipid homeostasis during the peripartal period [47,48]. Given that insulin resistance can impair glucose uptake and utilization by insulin-sensitive organs (e.g., adipose, muscle), thereby increasing reliance on gluconeogenesis and fatty acid oxidation, it is plausible that the observed changes reflect metabolic compensation rather than the direct effects of feeding RPM or RPC. Within this adaptive framework, the pentose phosphate pathway (PPP) also emerged as a key metabolic route in our analysis, particularly relevant for NADPH production [49], which is essential for maintaining cellular redox balance and the antioxidant function of glutathione [50]. RPM supplementation could enhance PPP flux by providing methyl groups via methionine metabolism, indirectly supporting NADPH production and glutathione-mediated antioxidant defense. An increased activity of this pathway in the peripartal period could contribute to the antioxidant response by the liver, especially in response to feeding RPM, which enhanced glutathione synthesis [6].

If RPM indeed modulates PPP activity, this could be mediated by its role in methylation reactions and phosphatidylcholine synthesis, indirectly supporting lipid metabolism and redox homeostasis [51,52]. Similarly, the phosphatidylinositol signaling pathway, which in nonruminants regulates glucose metabolism and cellular signaling [53], appeared overrepresented, especially with RPM (Table 1). Although in the bigger cohort of cows neither RPM nor RPC had an effect on plasma fatty acids or BHB, classical markers of energy balance, only RPC led to greater overall plasma glucose [22]. Notably, several metabolites including myo-inositol 1-phosphate, myo-inositol 4-phosphate, myo-inositol 6-phosphate, and D-myo-inositol 3-phosphate are involved in phosphoinositide metabolism. While these findings are intriguing, their exact role in hepatic metabolic regulation in response to feeding RPM remains speculative.

Additional evidence for altered one-carbon metabolism emerged from the observation of a trend toward greater overall concentrations of the M1172 molecular feature in the RPM and RPC groups compared with CON (Figure 6). This feature was tentatively annotated as 6-formylpterin (Table S2), an oxidized derivative of folate [54]. However, this identification is putative and has not been confirmed by authentic standards or MS/MS validation. Since methionine metabolism is closely linked to folate- and vitamin B_12_-dependent one-carbon metabolism, where folate donates methyl groups for the remethylation of homocysteine to methionine [55], this feature may reflect shifts in methylation dynamics during early lactation. In support of this possibility, WGCNA identified several molecular features in the turquoise module, which was positively correlated with the RPM and RPC groups (Figure 2). Among these, 2,4-dichlorophenol and 5-fluorodeoxyuridine monophosphate (5-FdUMP; M896 molecular feature) were of particular interest. 5-FdUMP is involved in folate metabolism [56] and may be indirectly linked to the methionine cycle through its role in one-carbon metabolism. Given the central role of folate metabolism in SAM synthesis, a key methyl donor involved in numerous biochemical reactions, including DNA methylation [57], this connection warrants further investigation. Similarly, 2,4-dichlorophenol (2,4-DCP), a compound influenced by hepatic detoxification in nonruminants [58], might be modulated by RPM supplementation, considering methionine’s role in glutathione synthesis and liver detoxification. While speculative, these findings raise the possibility that exogenous supply of dietary methionine and choline could influence the metabolism and clearance of 2,4-DCP.

Examining the top hub metabolites in the significant modules associated with RPM and RPC (Table 3; Figure 2), we identified M2125 (Figure 6) within the brown module, corresponding to SM(d18:1/20:1(11Z)) and SM(d18:2(4E,14Z)/20:0). Sphingomyelins (SMs) are functionally linked to methionine and choline metabolism due to their roles in lipid homeostasis and membrane structure [59,60]. Methionine and choline contribute to phospholipid synthesis via complementary pathways: methionine provides methyl groups through SAM for the methylation of phosphatidylethanolamine (PE) to phosphatidylcholine (PC), while choline acts as a direct precursor for PC formation via the Kennedy pathway [61,62,63]. Accordingly, changes in SM and PC-related metabolites likely reflect adaptive responses to RPM and RPC supplementation. Phosphatidylethanolamine (PE) derivatives, such as PE(18:2(9Z,12Z)/LTE4) and PE(LTE4/18:2(9Z,12Z)) (M2010; Figure 6), may also be influenced by methionine availability, as enhanced methylation to form PC could reduce PE levels due to preferential utilization. This aligns with the observed negative correlation between the brown module and the treated groups in the WGCNA analysis (Figure 2). Diacylglycerols (DGs), which serve as intermediates in phospholipid turnover, may also be affected: increased postruminal supply of methionine and choline may shift metabolism toward enhanced phospholipid biosynthesis, thereby reducing free DG pools. Similarly, SM levels could be affected by an increased demand for phospholipid remodeling, explaining their lower abundance in response to RPM and RPC [64,65,66].

In the blue module, which was positively correlated with RPM and particularly RPC (Figure 2), our MCC analysis identified N-Nervonoyl Tyrosine (M1034 molecular feature) as a notable compound potentially linked to hepatic function (Table 3 and Table 4). The metabolite N-Nervonoyl Tyrosine may be functionally associated with choline metabolism, given that it derives from nervonic acid, a monounsaturated fatty acid crucial for sphingolipid biosynthesis and myelin sheath integrity [67].

Overall, while methionine and choline enter phospholipid metabolism through distinct biochemical routes, the metabolomics profiles in liver largely reflected shared downstream adaptations in phospholipid remodeling rather than clearly separable and exclusive effects. These findings indicate overlapping metabolic responses with subtle treatment-specific nuances rather than discrete pathway-isolated effects.

Focusing on the metabolites significantly influenced by time (Table S3), the hepatic metabolome experienced marked shifts around parturition. These changes primarily involved carbohydrate metabolism, glycolysis/gluconeogenesis, fructose and mannose metabolism, galactose metabolism, and starch and sucrose metabolism (Table 2), indicating dynamic regulation of energy supply. Time-dependent alterations were also evident in amino acid pathways, including alanine, aspartate, and glutamate metabolism, as well as lysine degradation, suggesting adjustments in nitrogen metabolism and energy production in the liver. Furthermore, enrichment in phosphatidylinositol and inositol phosphate metabolism suggested alterations in hepatic signaling processes, while insulin resistance and glucagon signaling pathways reflected temporal modulation of hepatic glucose regulation. Collectively, these results support the view that liver metabolism undergoes coordinated temporal remodeling to meet energy demands during the periparturient period [68].

### 4.2. Insights on Skeletal Muscle Metabolism and Function Due to Feeding RPM and RPC

Methionine supplementation in dairy cows may contribute to muscle health by supporting protein synthesis and muscle integrity, particularly during the peripartal phase. As a precursor for the activation of the mTOR pathway, and although the extent of its role remains to be fully elucidated, methionine is thought to influence muscle protein synthesis under metabolic stress conditions [69]. Methionine is involved in creatine biosynthesis, which is important for muscle energy metabolism, and in glutathione production, potentially enhancing antioxidant defenses [70]. These combined functions could help protect muscle cells from oxidative damage and support muscle function, possibly mitigating the catabolic state during the transition period [71]. Similarly, choline might exert complementary effects on muscle function by contributing to membrane integrity and neuromuscular transmission. As a precursor to phosphatidylcholine, it has a structural role in stabilizing and maintaining muscle cell membranes [72]. Furthermore, since choline is essential for acetylcholine synthesis, a neurotransmitter involved in muscle contraction, it may play a role in sustaining neuromuscular function [73]. Besides its roles in structural and neurotransmitter events in muscle, choline may support muscle preservation indirectly through hepatic lipid metabolism. Evidence from the literature indicates that dietary choline supports hepatic lipid export via phosphatidylcholine synthesis and VLDL assembly, thereby reducing hepatic lipid accumulation and metabolic stress during the peripartal period [16,74]. This improved hepatic lipid handling may indirectly influence systemic amino acid utilization, potentially sparing methionine for other functions such as protein synthesis in muscle, although this mechanism is not directly demonstrated and remains speculative.

In our analysis, we observed a significantly altered abundance of several metabolites, including short-chain fatty acids (butyric acid, isobutyric acid, acetoin, methyl and ethyl esters) and amino acid derivatives such as iodotyrosine and perflutren (Table 5), suggesting that supplementation modulated both energy and amino acid metabolism in muscle. Complementary evidence emerged from the WGCNA analysis, where several hub compounds of the black module (Figure 5; Table 6 and Table S7) were identified that could be closely linked to feeding RPM, particularly through their involvement in glutathione metabolism [75]. Notably, the M1409 molecular feature was associated with pyroglutamic acid and 1-pyrroline-4-hydroxy-2-carboxylate (Table S7). Pyroglutamic acid (also known as 5-oxoproline) is a critical component of the γ-glutamyl cycle, which plays a vital role in glutathione production and maintaining cellular redox balance [76]. The metabolite 1-pyrroline-4-hydroxy-2-carboxylate is an intermediate in proline metabolism [77], which is tightly linked to glutathione metabolism and oxidative stress regulation [78]. Furthermore, the detection of cis-2-methylaconitate suggests that RPM supplementation may modulate mitochondrial energy metabolism, supporting both the energetic and antioxidant demands of skeletal muscle during the transition period. Taken together, these findings suggest that feeding RPM may enhance antioxidant capacity and energy metabolism in muscle, although further studies are needed to confirm these tissue-specific adaptations.

Time significantly influenced the skeletal muscle metabolome, with several metabolites demonstrating dynamic changes across the transition period (Table S6). Notably, we observed fluctuations in pyridoxal 5′-phosphate (active vitamin B6) [79], thiamine pyrophosphate [80], and dehydroascorbic acid, which reflect adjustments in cofactor availability and antioxidant capacity during the peripartal phase. Alterations in intermediates of the TCA cycle, including cis- and trans-aconitic acid, as well as isocitric acid lactone, suggest a temporal remodeling of mitochondrial energy metabolism, potentially to meet the fluctuating energetic demands of muscle. The detection of amino acid derivatives such as selenocysteine, allysine [81], and N-methylhydroxyproline [82] suggests time-dependent changes potentially linked to protein turnover, collagen remodeling, and redox-related processes. Collectively, these results indicate that the temporal effect on skeletal muscle involves the modulation of metabolic and structural pathways consistent with physiological adaptations occurring during the transition period.

## 5. Conclusions

Feeding RPM and RPC during the peripartal period exerts a complementary influence on the metabolism and function of liver and skeletal muscle. In the liver, feeding both methyl donors was associated with changes in key pathways related to glucose and lipid metabolism, including glycolysis/gluconeogenesis, the pentose phosphate pathway, and inositol metabolism, contributing to metabolic flexibility and systemic energy homeostasis. Alterations in glycolytic intermediates, folate derivatives, and lipid-related metabolites point to potential shifts in glucose production, one-carbon metabolism, and membrane dynamics. In skeletal muscle, RPM was linked with metabolites potentially related to protein synthesis, glutathione-dependent antioxidant defenses, and mitochondrial metabolism, while choline appeared to influence compounds associated with membrane stability and neuromuscular function. Metabolomics and WGCNA analyses highlighted alterations in short-chain fatty acids, amino acid derivatives, and key hub metabolites, suggesting tissue-specific metabolic adaptations. Temporal effects around parturition were also evident in both liver and muscle, particularly affecting carbohydrate, amino acid, and redox-related metabolism, consistent with physiological adjustments required during the transition phase. Collectively, these results reinforce the importance of targeted nutritional strategies, such as RPM and RPC supplementation, in supporting metabolic health and antioxidant capacity in dairy cows during the transition period.

## Figures and Tables

**Table 1:** Biological KEGG terms revealed from the enrichment analysis (FDR ≤ 5%) of the 104 metabolites associated with the treatment effect in liver performed using MBRole3.

KEGG Pathway	Set	In Set	Background	In Background	*p*-Value	FDR
Fructose and mannose metabolism	19	6	4268	54	<0.000	<0.000
Inositol phosphate metabolism	19	4	4268	47	<0.000	0.001
Galactose metabolism	19	4	4268	46	<0.000	0.001
Amino sugar and nucleotide sugar metabolism	19	5	4268	108	<0.000	0.001
Glucagon signaling pathway	19	3	4268	26	<0.000	0.001
Phosphatidylinositol signaling system	19	3	4268	29	<0.000	0.001
Glycolysis/Gluconeogenesis	19	3	4268	31	<0.000	0.002
Starch and sucrose metabolism	19	3	4268	37	0.001	0.002
Insulin resistance	19	2	4268	19	0.003	0.012
AMPK signaling pathway	19	2	4268	22	0.004	0.014
Metabolic pathways	19	18	4268	2867	0.005	0.017
Diabetic cardiomyopathy	19	2	4268	39	0.013	0.027
Phenylalanine, tyrosine and tryptophan biosynthesis	19	2	4268	34	0.010	0.027
Central carbon metabolism in cancer	19	2	4268	37	0.011	0.027
Autophagy—other	19	1	4268	3	0.013	0.027
Carbon metabolism	19	3	4268	114	0.013	0.027
Pentose phosphate pathway	19	2	4268	35	0.010	0.027
Glycosylphosphatidylinositol (GPI)-anchor biosynthesis	19	1	4268	4	0.018	0.033
Lysosome	19	1	4268	4	0.018	0.033
Kaposi sarcoma-associated herpesvirus infection	19	1	4268	5	0.022	0.039
Autophagy—animal	19	1	4268	6	0.026	0.044

**Table 2:** Biological KEGG terms revealed from the enrichment analysis (FDR ≤ 5%) of the 552 metabolites associated with the time effect in liver performed using MBRole3.

KEGG Pathway	Set	In Set	Background	In Background	*p*-Value	FDR
Fructose and mannose metabolism	51	6	4268	54	<0.000	0.001
Amino sugar and nucleotide sugar metabolism	51	8	4268	108	<0.000	0.001
Glycolysis/Gluconeogenesis	51	4	4268	31	<0.000	0.007
Metabolic pathways	51	45	4268	2867	<0.000	0.007
Insulin resistance	51	3	4268	19	0.001	0.016
Galactose metabolism	51	4	4268	46	0.002	0.019
Inositol phosphate metabolism	51	4	4268	47	0.002	0.019
Lysine degradation	51	4	4268	50	0.003	0.021
Glucagon signaling pathway	51	3	4268	26	0.003	0.023
Alanine, aspartate and glutamate metabolism	51	3	4268	28	0.004	0.026
Phosphatidylinositol signaling system	51	3	4268	29	0.005	0.026
Glyoxylate and dicarboxylate metabolism	51	4	4268	62	0.006	0.030
Starch and sucrose metabolism	51	3	4268	37	0.009	0.043
Diabetic cardiomyopathy	51	3	4268	39	0.011	0.047

**Table 3:** Top 10 ranked metabolites in key modules identified using the Maximal Clique Centrality (MCC) approach in Cytoscape for the liver dataset. Columns labeled turquoise, blue, and brown correspond to the color-coded modules identified by WGCNA. Molecular features highlighted in bold and underlined were consistently identified as hub metabolites by both the MCC and Module Membership–Gene Significance approaches.

Liver Dataset WGCNA	Turquoise Module	Blue Module	Brown Module
**Top 10 hub** **metabolites**	M261	M28	**M2010**
	M394	M1085	M1749
	M251	**M843**	**M2021**
	M396	M836	M2241
	M117	M4	M2125
	M257	M1123	M1734
	M12	M902	M1967
	M258	M137	M2014
	M471	M10	M2174
	M15	M1034	M2186

**Table 4:** Molecular features differentially highlighted (uncorrected *p*-value ≤ 0.01) in skeletal muscle from multiparous Holstein cows fed a control diet (CON), or CON plus rumen-protected methionine (RPM) or rumen-protected choline (RPC) during the close-up (from −10 d to parturition) and early lactation (from +7 to +20 days in milk) periods. Each treatment group included 7 cows per treatment at each time point, totaling 21 samples per treatment. Data units are log_10_ and autoscaled, as processed using MetaboAnalyst v6.0. SEM stands for standard error of the mean.

		Treatment				*p*-Value	
	CON	RPM	RPC	SEM	TRT	TIME	TRT × TIME
Total (no. of cows)	21	21	21				
Metabolite							
M137	−0.106	0.137	−0.031	0.028	0.007	0.004	0.854
M141	−0.069	0.017	0.052	0.016	0.004	0.000	0.001
M142	−0.071	0.017	0.054	0.016	0.004	0.000	0.001
M145	−0.204	0.165	0.040	0.046	0.002	0.610	0.642
M157	−0.086	0.112	−0.026	0.030	0.006	0.000	0.320
M256	−0.070	0.017	0.053	0.016	0.004	0.000	0.001
M339	0.154	−0.364	0.210	0.077	0.004	0.700	0.906
M395	−0.327	0.335	−0.008	0.069	0.001	0.030	0.716
M438	−0.226	0.321	−0.094	0.069	0.004	0.010	0.906
M557	−0.297	0.150	0.147	0.074	0.003	0.593	0.796
M560	0.197	0.111	−0.308	0.065	0.009	0.807	0.603
M671	−0.059	−0.237	0.296	0.083	0.004	0.146	0.914
M738	0.040	−0.224	0.183	0.067	0.002	0.015	0.351
M748	−0.501	0.052	0.449	0.120	0.009	0.015	0.376
M868	0.557	−0.145	−0.412	0.132	0.010	0.056	0.679
M952	0.116	−0.273	0.157	0.090	0.008	0.000	0.061
M975	0.567	−0.362	−0.205	0.132	0.001	0.319	0.335
M1003	0.569	−0.017	−0.553	0.146	0.001	0.213	0.628
M1032	−0.114	−0.413	0.527	0.124	0.004	0.120	0.519
M1033	−0.477	0.032	0.446	0.120	0.008	0.058	0.627
M1040	0.597	−0.194	−0.402	0.137	0.001	0.177	0.723
M1044	0.254	−0.694	0.439	0.157	0.010	0.000	0.846
M1093	0.213	0.332	−0.545	0.141	0.009	0.162	0.846
M1252	−0.497	0.296	0.201	0.106	0.003	0.188	0.938
M1303	−0.096	−0.439	0.535	0.084	0.000	0.365	0.459
M1328	−0.582	−0.076	0.658	0.163	0.004	0.118	0.440
M1399	−0.330	−0.116	0.446	0.132	0.000	0.000	0.001
M1400	−0.341	0.487	−0.145	0.125	0.008	0.439	0.548
M1401	0.375	−0.182	−0.193	0.098	0.004	0.054	0.663
M1415	0.473	0.053	−0.526	0.140	0.005	0.003	0.003
M1428	−0.254	−0.122	0.376	0.128	0.004	0.000	0.380

**Table 5:** Annotated hits in muscle obtained by manual curation and the R package MetaboAnnotation, after searching against the HMDB library using the average molecular weight (AMW) with a tolerance of ±0.005 Da of the molecular features differentially highlighted (*p*-value ≤ 0.01). This analysis pertains to the overall treatment effect of rumen-protected methionine and rumen-protected choline supplementation. Muscle tissue was harvested from multiparous Holstein cows fed rumen-protected methionine or rumen-protected choline during the close-up (from −10 d to parturition) and early lactation (from +7 to +20 days in milk) periods.

Molecular Feature				Match on HMDB		
	Mass Weight	Mass-to-Charge Ratio	Retention Time	HMDB ID	Name	AVM
M1003	188.0165582	189.0238342	6.439240456	HMDB0014696	Perflutren	188.0193
M1040	307.0827203	308.0899963	4.604357719	HMDB0000021	Iodotyrosine	307.0851
M157	132.0894342	133.0967102	3.246370316	HMDB0060402	5.6-Dihydro-5-fluorouracil	132.0931
M395	88.10029846	89.10757446	3.200959682	HMDB0000039	Butyric acid	88.1051
				HMDB0001873	Isobutyric acid	88.1051
				HMDB0003243	Acetoin	88.1051
				HMDB0030062	Methyl propionate	88.1051
				HMDB0031217	Ethyl acetate	88.1051
				HMDB0031507	1-Hydroxy-2-butanone	88.1051
				HMDB0040253	Propyl formate	88.1051
				HMDB0040579	Isopropyl formate	88.1051
				HMDB0244216	Dioxane	88.1051
				HMDB0303161	(R)-Acetoin	88.1051
M438	71.07374446	89.10757446	3.194950104	HMDB0004296	Acrylamide	71.0779
				HMDB0245904	3-Hydroxypropionitrile	71.0779

**Table 6:** Top 10 hub metabolites in the black module of the muscle dataset, identified based on network connectivity using the Maximal Clique Centrality (MCC) approach in Cytoscape. Hub metabolites are defined as highly connected nodes within the module. HMDB annotations are provided.

Molecular Feature				Match on HMDB		
	Mass Weight	Mass-to-Charge Ratio	Retention Time	HMDB ID	Name	AVM
M1135	249.1696039	250.1768799	18.5199928	HMDB0258136	2-[(2S)-2-Amino-3-carboxypropanoyl]oxybutanedioic acid	249.1750
M1385	126.0426431	127.0499191	11.0106058	HMDB0012228	Ethylphosphate	126.0483
				HMDB0061734	Dimethylphosphate	126.0480
				HMDB0252055	Ethyl methylphosphonofluoridate	126.0670
				HMDB0252413	Fluroxene	126.0780
M1388	271.1047845	272.1120605	10.8680191	HMDB0256151	8-Quinolinol, 5,7-dichloro-2-((dimethylamino)methyl)-	271.1400
				HMDB0256293	Pentrinitrol	271.1380
				HMDB0304535	Alpha-D-glucuronate 1-phosphate	271.0950
M1409	129.0785852	130.0858612	4.3066320	HMDB0000267	Pyroglutamic acid	129.1140
				HMDB0001369	Pyrroline hydroxycarboxylic acid	129.1140
				HMDB0001843	N-Acryloylglycine	129.1140
				HMDB0002234	1-Pyrroline-4-hydroxy-2-carboxylate	129.1140
				HMDB0015231	Flucytosine	129.0925
				HMDB0041861	Cyanuric acid	129.0742
				HMDB0061093	Dimethadione	129.1140
				HMDB0062585	(3R,5S)-1-Pyrroline-3-hydroxy-5-carboxylic acid	129.1150
				HMDB0244988	5-Hydroxy-2-imino-1-methylimidazolidin-4-one	129.1190
				HMDB0245148	3-Hydroxy-1-methylpyrrolidine-2,5-dione	129.1150
				HMDB0245451	2,4-Difluoroaniline	129.1100
				HMDB0246561	4-Oxo-L-proline	129.1150
				HMDB0258122	(2S)-3,4-Dioxoazetidine-2-carboxylic acid	129.0710
				HMDB0260147	(2S)-6-Oxa-1-azabicyclo [3.1.0]hexane-2-carboxylic acid	129.1150
M1427	188.0081812	189.0154572	3.7899945	HMDB0014696	Perflutren	188.0193
M1441	188.1269250	189.1342010	3.6929231	HMDB0006357	cis-2-Methylaconitate	188.1348
				HMDB0060320	(Z)-But-1-ene-1,2,4-tricarboxylate	188.1348
				HMDB0243872	1-Ethoxymethyl-5-fluorouracil	188.1580
				HMDB0304057	2-Amino-3,7-dideoxy-D-threo-hept-6-ulosonate	188.1590
M981	123.5269418	124.5342178	11.2667351	HMDB0243713	3-Chloro-D-alanine	123.5400
M1341	133.015307	134.022583	4.64839411	-	-	-
M1390	298.0293756	299.0366516	4.64576054	-	-	-
M1377	160.0042902	161.0115662	4.67561293	-	-	-

## Data Availability

The data supporting the conclusions of this article will be made available by the authors on request.

## Supplementary Materials

The following supporting information can be downloaded at: https://www.mdpi.com/article/10.3390/ani16050705/s1, Figure S1. The left panel shows the scale-free fit index versus soft-thresholding power in liver dataset. The right panel displays the mean connectivity versus soft-thresholding power. Power 3 was chosen, for which the fit index curve flattens out upon reaching a high value (>0.9) while balancing mean connectivity drop; Figure S2. Cluster dendrogram of metabolites in the liver dataset generated by WGCNA. Branches correspond to individual metabolites, and colors below the dendrogram indicate module assignment. After module merging, nine distinct modules were identified. Metabolites not assigned to any module are shown in grey; Figure S3. The left panel shows the scale-free fit index versus soft-thresholding power in muscle dataset. The right panel displays the mean connectivity versus soft-thresholding power. Power 3 was chosen, for which the fit index curve flattens out upon reaching a high value (>0.9) while balancing mean connectivity drop; Figure S4. Cluster dendrogram of metabolites in the muscle dataset generated by WGCNA. Branches correspond to individual metabolites, and colors below the dendrogram indicate module assignment. After module merging, nine distinct modules were identified. Metabolites not assigned to any module are shown in grey; Table S1. Molecular features differentially highlighted (FDR ≤ 0.05) in liver from multiparous Holstein cows (n = 21 per treatment across time points) fed a control diet (CON), or CON plus rumen-protected methionine (RPM) or rumen-protected choline (RPC) during the close-up (from −10 d to parturition) and early lactation (from +7 to +20 days in milk) periods. Data units are log_10_ and autoscaled, as processed using MetaboAnalyst v6.0. SEM stands for standard error of the mean. Molecular features corresponding to metabolites identified as top VIPs in the sPLS-DA analysis and consistently detected are highlighted in bold and underlined. Table S2. Annotated hits in liver obtained by manual curation and the R package MetaboAnnotation, after searching against the HMDB library using the average molecular weight (AMW) with a tolerance of ±0.005 Da of 140 molecular features differentially highlighted (FDR ≤ 0.05). Liver tissue was harvested from multiparous Holstein cows fed rumen-protected methionine or rumen-protected choline during the close-up (from −10 d to parturition) and early lactation (from +7 to +20 days in milk) periods; Table S3. Annotated hits obtained by manual curation and the R package MetaboAnnotation, after searching against the HMDB library using the average molecular weight (AMW) with a tolerance of ±0.005 Da, of 1586 metabolites differentially highlighted (FDR ≤ 0.05) in liver from multiparous Holstein cows fed rumen-protected methionine and rumen-protected choline during the close-up (from −10 d to parturition) and early lactation (from +7 to +20 days in milk) periods due to time effect; Table S4. Annotated hits in liver obtained by manual curation and the R package MetaboAnnotation, after searching against the HMDB library using the average molecular weight (AMW) with a tolerance of ±0.05 Da, of the top 10 ranked molecular features in four candidate modules identified using the Maximal Clique Centrality (MCC) approach in Cytoscape. Liver tissue was harvested from multiparous Holstein cows fed rumen-protected methionine (RPM) and rumen-protected choline (RPC) during the close-up (from −10 d to parturition) and early lactation (from +7 to +20 days in milk) periods. This analysis pertains to the overall treatment effect rumen-protected methionine and rumen-protected choline supplementation. Molecular features highlighted in bold and underlined were consistently identified as hub metabolites by both the MCC and Module Membership–Gene Significance approaches; Table S5. Molecular features differentially highlighted (FDR ≤ 0.05) in muscle from multiparous Holstein cows (n = 21 per treatment across time points) fed rumen-protected methionine (RPM) and rumen-protected choline (RPC) during the close-up (from −10 d to parturition) and early lactation (from +7 to +20 days in milk) periods due to time (TIME) effect. CON stands for control group. SEM stands for standard error of the mean; Table S6. Annotated hits in muscle obtained by manual curation and the R package MetaboAnnotation, after searching against the HMDB library using the average molecular weight (AMW) with a tolerance of ±0.005 Da of molecular features differentially highlighted (FDR ≤ 0.05) due to time effect. Muscle tissue was harvested from multiparous Holstein cows fed rumen-protected methionine or rumen-protected choline during the close-up (from −10 d to parturition) and early lactation (from +7 to +20 days in milk) periods; Table S7. Annotated hits in muscle obtained by manual curation and the R package MetaboAnnotation, after searching against the HMDB library using the average molecular weight (AMW) with a tolerance of ±0.05 Da, of the top 10 ranked metabolites in key modules identified using the Maximal Clique Centrality approach in Cytoscape. Muscle tissue was harvested from multiparous Holstein cows fed rumen-protected methionine or rumen-protected choline during the close-up (from −10 d to parturition) and early lactation (from +7 to +20 days in milk) periods due to treatment effect.

## Abbreviations

The following abbreviations are used in this manuscript:

β	Soft threshold power
BER	Balanced error rate
CON	Control
DGs	Diacylglycerols
FDR	False discovery rate
GS	Gene significance
HMDB	The Human Metabolome Database
IDs	Identifiers
KEGG	Kyoto Encyclopedia of Genes and Genomes
MCC	Maximal clique centrality
MEDissThres	Module dissimilarity threshold
MM	Module membership
MS-DIAL	Mass Spectrometry Data Independent Analysis
PC	Phosphatidylcholine
PE	Phosphatidylethanolamine
PERMANOVA	Permutational Multivariate Analysis Of Variance
PLS-DA	Partial Least Squares Discriminant Analysis
REA	ReaShure
RPC	Rumen-protected choline
RPLC	Reversed-phase liquid chromatography
RPM	Rumen-protected methionine
SAM	S-adenosylmethionine
SM	Sphingomyelin
SMA	Smartamine
UHLC	Ultra-high performance liquid chromatography
VIP	Variable importance in projection
VLDL	Very-low-density lipoprotein
WGCNA	Weighted gene co-expression network analysis
