# Supplementary material for: Liver and Skeletal Muscle Metabolome Characterization in Peripartal Dairy Cows Fed Rumen-Protected Methionine or Rumen-Protected Choline
Source: Animals (Basel). 2026 Feb 24;16(5):705. doi: 10.3390/ani16050705 (PMC12983954; doi:10.3390/ani16050705)
Supplement: Supplementary file 1 [file animals-16-00705-s001.zip › animals-4140613-SuppFigs_v2.pdf]

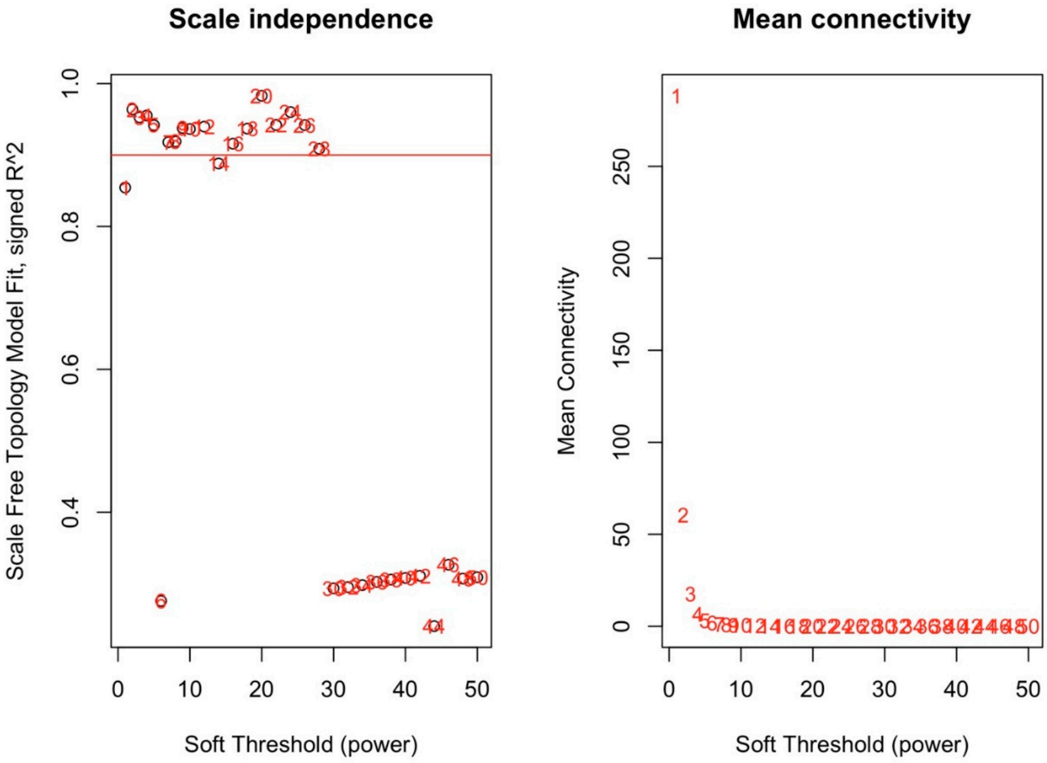


**Figure S1.** The left panel shows the scale-free fit index versus soft-thresholding power in liver dataset. The right panel displays the mean connectivity versus soft-thresholding power. Power 3 was chosen, for which the fit index curve flattens out upon reaching a high value (> 0.9) while balancing mean connectivity drop.

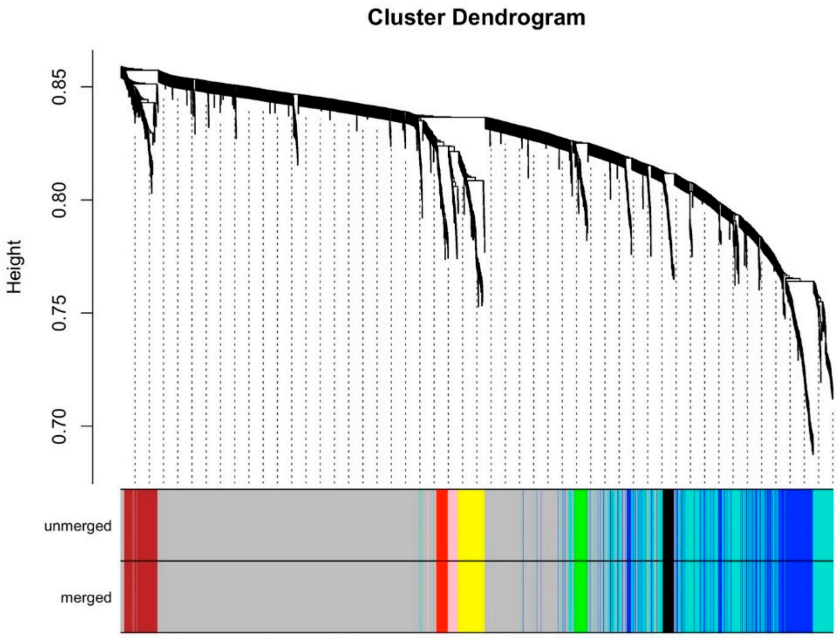


**Figure S2.** Cluster dendrogram of metabolites in the liver dataset generated by WGCNA. Branches correspond to individual metabolites, and colors below the dendrogram indicate module assignment. After module merging, nine distinct modules were identified. Metabolites not assigned to any module are shown in grey.

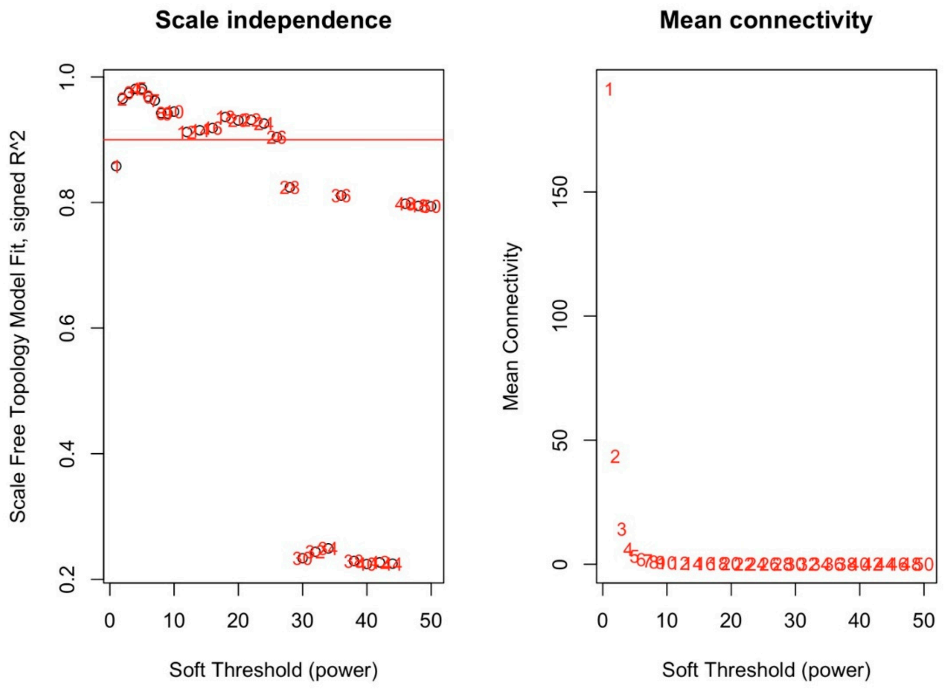


**Figure S3.** The left panel shows the scale-free fit index versus soft-thresholding power in muscle dataset. The right panel displays the mean connectivity versus soft-thresholding power. Power 3 was chosen, for which the fit index curve flattens out upon reaching a high value (> 0.9) while balancing mean connectivity drop.

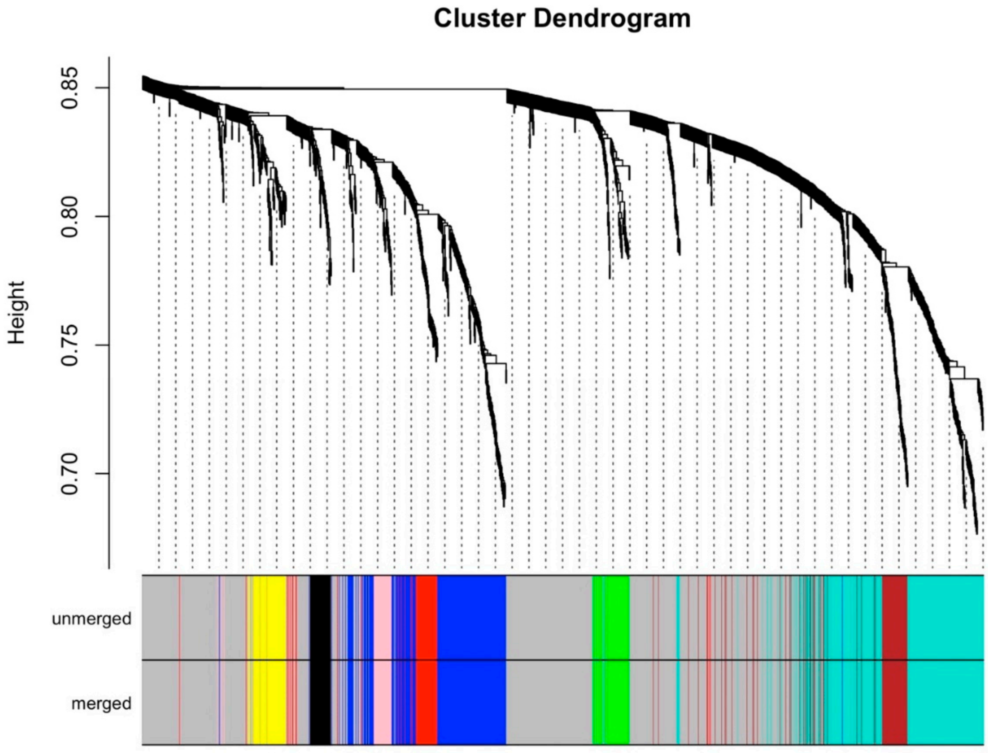


**Figure S4.** Cluster dendrogram of metabolites in the muscle dataset generated by WGCNA. Branches correspond to individual metabolites, and colors below the dendrogram indicate module assignment. After module merging, nine distinct modules were identified. Metabolites not assigned to any module are shown in grey.
